# Predictors of the Survival of Primary and Secondary Older Osteosarcoma Patients

**DOI:** 10.7150/jca.32627

**Published:** 2019-08-07

**Authors:** Zhan Wang, Bo Wu, Yuanxi Zhou, Xin Huang, Weibo Pan, Meng Liu, Xiaobo Yan, Nong Lin, Zhaoming Ye

**Affiliations:** Department of Orthopaedics, Centre for Orthopaedic Research, Orthopedics Research Institute of Zhejiang University, The Second Affiliated Hospital, Zhejiang University School of Medicine, 88 Jiefang Road, Hangzhou, Zhejiang 310000, P.R. China

**Keywords:** primary osteosarcoma, secondary osteosarcoma, adults, prognostic factor, therapy

## Abstract

**Purpose:** Older osteosarcoma patients have a very poor prognosis and treatment for them remains a challenge. The outcomes and potential prognostic factors of primary or secondary older osteosarcoma patients are rarely documented. Therefore, we examined the prognosis of the two special cohorts to identify possible prognostic factors, and provide optimal treatment strategy for them.

**Methods:** The Surveillance, Epidemiology, and End Results (SEER) program database was used to identify osteosarcoma patients aged over 40 years from 1973 to 2015. The prognostic analysis was performed using the Kaplan-Meier method and a Cox proportional hazards regression model.

**Results:** In total, 1162 primary older osteosarcoma patients and 444 secondary older osteosarcoma patients were eligible for this study. The OS and CSS rates of the primary older osteosarcoma patients at 5-year were 38.5% and 37.1%, respectively. The 3- and 5-year OS rates of the secondary older osteosarcoma patients were 22.8% and 14.6%, respectively. On multivariate analysis of the primary older osteosarcoma patients, age > 60, male, axial site, high grade, metastasis, tumor size>10 cm, no surgery, and radiation treatment were negatively associated with OS. In terms of CSS, age, gender, decade of diagnosis, tumor site, tumor grade, tumor stage, tumor size, and surgery were independent prognostic factors. A multivariate Cox regression model showed that secondary older osteosarcoma patients of high grade, metastasis, tumor size > 10 cm, no surgery, and no chemotherapy were independent predictors of decreased OS.

**Conclusions:** Surgery in combination with chemotherapy should be recommended for the treatment of the secondary older osteosarcoma patients, while for the primary older osteosarcoma patients, only surgery should be recommended.

## Introduction

Osteosarcoma is the most common type of primary bone sarcomas and occurs predominantly in children and young adults 1. The demographic, prognostic, and outcome data of osteosarcoma in children and young adults, or patients of all ages are well documented 2-4. Current standard treatment of younger osteosarcoma patients includes preoperative chemotherapy, surgical resection of all tumor and postoperative chemotherapy 5. The 10-year cancer specific survival (CSS) rate for patients with localized/regional osteosarcoma is approximately 70%, while for patients with metastatic disease is 24% 3. Age at diagnosis, tumor size and site, pathological fractures, tumor grade, metastasis at presentation, and systemic chemotherapy are all associated with the prognosis of osteosarcoma 2, 3, 6, 7.

The second incidence peak of primary osteosarcoma is in the older. Coincidentally, secondary osteosarcoma also frequently occurs in the older persons mainly due to Paget's disease or post-radiation and usually has a much poorer outcome compared to primary osteosarcoma 8, 9. Older osteosarcoma patients often have a worse prognosis compared with younger patients 7, 10. Recently, Iwata *et al*. 10 reported poor prognoses in 86 elderly osteosarcoma patients, with a 5-year overall survival rate 38.8%. They found that tumor site, metastasis at diagnosis, surgery, and surgical margins were significantly associated with survival, whereas chemotherapy had no influence on survival. However, some studies reported that aggressive treatment with chemotherapy can offer a favorable outcome for older osteosarcoma patients 11-13. Although osteosarcoma is radioresistant, some studies have reported that radiotherapy is an effect adjuvant treatment for local control 14, 15. Older osteosarcoma patients may receive radiotherapy due to its lower toxicity compared with systemic chemotherapy 13, 15. The effects of radiotherapy on survival of older osteosarcoma patients are rarely studied. Therefore, this study also assessed the prognostic utility of radiotherapy in older osteosarcoma patients.

Optimal treatment for older osteosarcoma patients is still controversial. To obtain deeper insight into primary and secondary older osteosarcoma patients, we analyzed osteosarcoma patients aged over 40 from 1973 to 2015 in the Surveillance, Epidemiology, and End Results (SEER) program database of the National Cancer Institute. This was a large-scale study of older osteosarcoma patients that aimed to confirm the predictors of survival.

## Materials and Methods

### Patient population

A total of 6224 patients diagnosed with osteosarcoma of bone were identified from 1973 to 2015. All patient data were obtained using the case-listing session procedure from the SEER program database. The database is publicly available and does not include unique patient identifiers. This study followed standard guidelines and was approved by the Ethics Committee of the Second Affiliated Hospital of Zhejiang University School of Medicine.

**Figure 1:**
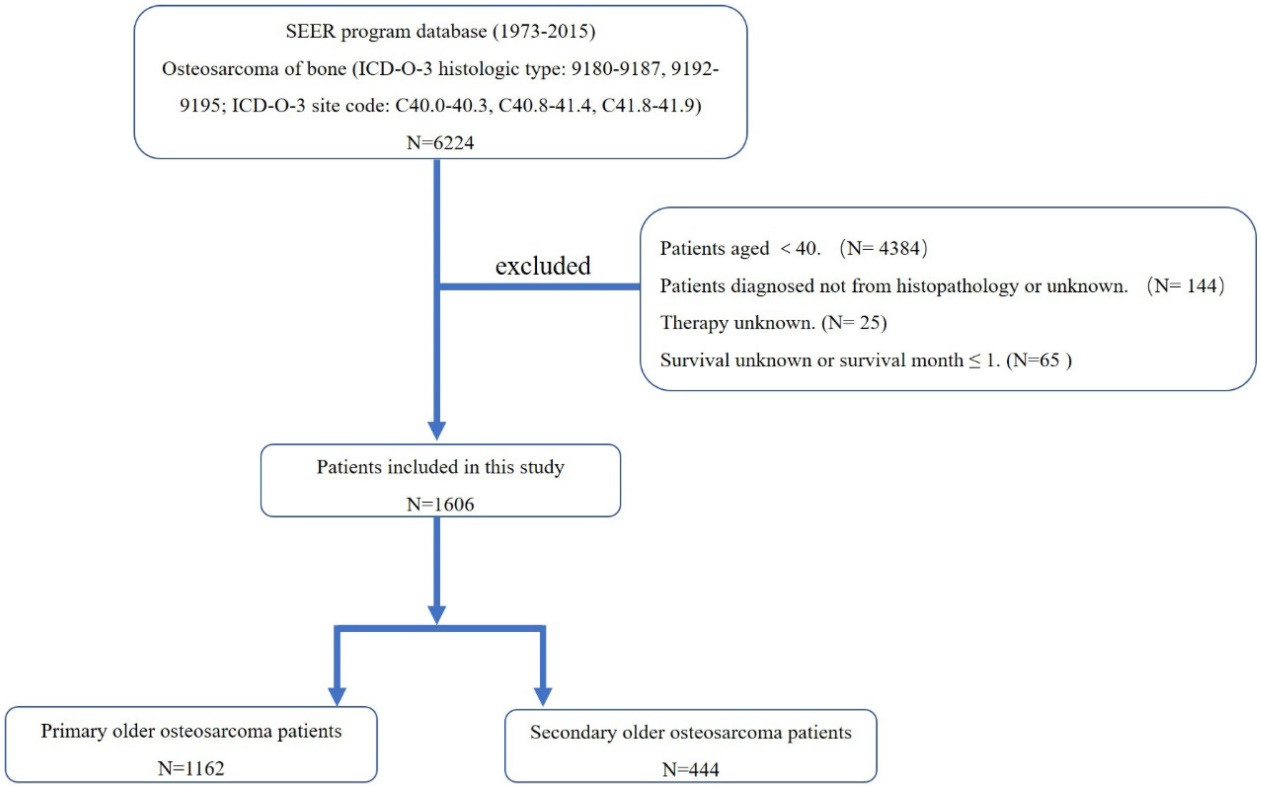
The flow chart for selection of study population. (Abbreviations: SEER, Surveillance, Epidemiology, and End Results; ICD-O-3, international classification of diseases for oncology, 3rd edition.)

First, the International Classification of Diseases for Oncology, 3^rd^ edition (ICD-O-3) was used to identify patients with osteosarcoma of bone (ICD-O-3 histologic type: 9180-9187, 9192-9195; ICD-O-3 site code: C40.0-40.3, C40.8-41.4, C41.8-41.9), using the case-listing procedure. Only patients aged over 40 were enrolled, by reference to the age at diagnosis. Previous researches also recognized osteosarcoma patients aged over 40 as older osteosarcoma patients. All patients were confirmed histologically, based either on biopsy results or the surgical specimen. One hundred and forty-four patients diagnosed only on the basis of the clinical presentation, or according to the radiography were excluded. Twenty-five patients with unknown therapy were excluded. Sixty-five patients with missing survival information or survival month ≤ 1 were also excluded. The sequence number was used to classify older patients as having a primary osteosarcoma without any other primary malignant tumors (sequence number ≤ 1) (n =1162) and as having osteosarcoma as a secondary malignancy (sequence number ≥ 2) (n = 444) (Figure. 1). Data extracted from the SEER database included age, gender, race, year of diagnosis, location, tumor grade, tumor stage, tumor type, tumor size, surgical treatment, radiation treatment, chemotherapy, cause of death, and survival time. Surgery or radiation treatment for tumors in our study refers to treatment for local primary tumors. We divided the location into three categories: (1) appendicular (long and short bones of the upper and lower extremities), (2) axial (pelvis and spine), and (3) other locations (mandible, skull, rib, sternum, clavicle, and other atypical locations). Given that age is an important predictor for survival of osteosarcoma, we further divided older osteosarcoma patients into two categories ( ≤ 60 group and >60 group) according to the mean and median age at diagnosis.

### Statistical methods

The statistical analyses were performed using Microsoft Excel 2016 (Microsoft Corp., Redmond, WA, USA) and SPSS software (ver. 21.0; SPSS Inc., Chicago, IL, USA). Overall survival (OS) was defined as the time from diagnosis to death from any cause and cancer-specific survival (CSS) was regarded as the time from diagnosis to death due specifically to cancer. The Kaplan-Meier method was used to draw the OS and CSS curves and calculate median survival. Observations were censored if the patient was alive at the time of the last follow-up. Univariate analyses were performed using the Kaplan-Meier method with the log-rank test. Multivariate analysis was used to determine the independent predictors of OS and CSS with a Cox proportional hazards regression model. The hazard ratios (HRs) and corresponding 95% confidence intervals (CIs) were used to show the effect of various factors on OS and CSS. Differences were deemed statistically significant if the P-value was less than 0.05.

## Results

### Demographic and clinical characteristics of osteosarcoma patients aged over 40

In total, 1606 patients were eligible for our study, including 1162 primary older osteosarcoma patients and 444 secondary older osteosarcoma patients. In primary older osteosarcoma cohort, the mean and median patient age at diagnosis were 58 and 56 years, respectively. In terms of location, 54.8% tumors were located in the extremities, 19.4% in the axial skeleton, and 25.8% in other sites. Histologically, 13.6% of the cases were low grade, and 52.7% were high grade. The majority of the patients were diagnosed as osteosarcoma, not otherwise specified (NOS) (71.9%). Information on the tumor size was available in 62.3% cases, and was categorized into three groups. More than two thirds of the patients (75.5%) received local surgery, 272 patients (23.4%) received radiation treatment, and about half of patients had chemotherapy. Ultimately, 781 patients (67.2%) died, of whom 631 died of cancer. The 3- and 5-year OS rates of the entire cohort were 47.1% and 38.5%, respectively. The 3- and 5-year CSS rates were 45.6% and 37.1%, respectively (Table 1). The median OS and CSS were 30.0±2.6 and 28.0±2.4 months, respectively, suggesting a poor prognosis for this cohort (Table 2).

In secondary older osteosarcoma cohort, the mean and median patient age at diagnosis were 66 and 67 years, respectively. Compared with the primary older osteosarcoma, secondary older osteosarcoma shows a predilection for axial sites (33.8%). Histologically, 6.8% of the cases were low grade, and 59.0% were high grade. The majority of the cases were also osteosarcoma, not otherwise specified (NOS) (65.5%). Information on the tumor size was available in 53.2% cases, and was categorized into three groups. More than half of the patients (64.9%) received local surgery, 104 patients (23.4%) received radiation treatment, and about half of patients had chemotherapy. The 3- and 5-year OS rates of this cohort were 22.8% and 14.6%, respectively (Table 1). The median OS of this population was 12.0 ± 0.9, suggesting a poorer prognosis than primary older osteosarcoma patients (Table 2).

### Univariate analyses of variables associated with OS or CSS in osteosarcoma patients aged over 40

Univariate analyses of variables associated with survival of older osteosarcoma patients are shown in Table 3. Among primary older osteosarcoma patients, univariate analyses revealed that race was not associated with either OS or CSS. Female patients had significantly better outcomes than male patients, with a longer median survival. OS and CSS showed a statistically significant difference in survival based on decade of diagnosis. Patients with axial tumor location had worse outcomes than those with appendicular or other tumor locations. Both OS and CSS differed significantly with tumor grade, with a high tumor grade portending a worse prognosis.

**Figure 2:**
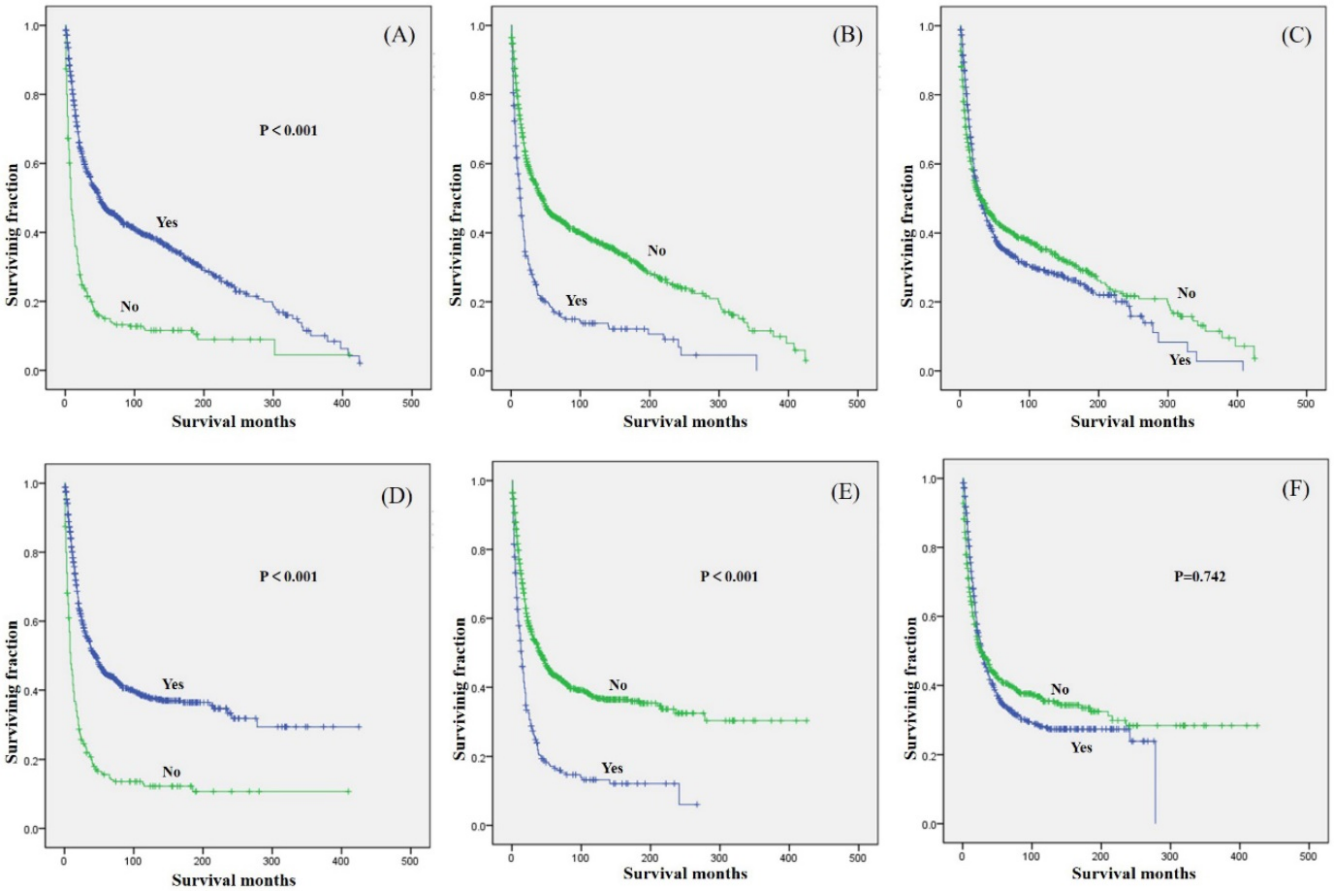
Kaplan-Meier method estimated OS and CSS in primary osteosarcoma patients aged over 40 years. OS stratified by (A) surgery, (B) radiation, and (C) chemotherapy. CSS stratified by (D) surgery, (E) radiation, and (F) chemotherapy.

Tumor stage was associated with significant differences in OS and CSS, with metastasis predicting a worse prognosis. Similarly, tumor size was associated with significant differences in OS and CSS, with smaller tumor size predicting a better prognosis. In terms of treatment, patients who underwent surgical treatment had better OS and CSS than those who did not (Fig.2A and 2D). However, patients who received radiation treatment had worse OS and CSS than those who did not (Fig.2B and 2E). No significant difference in OS or CSS, based on chemotherapy was observed (Fig.2C and 2F).

**Figure 3:**
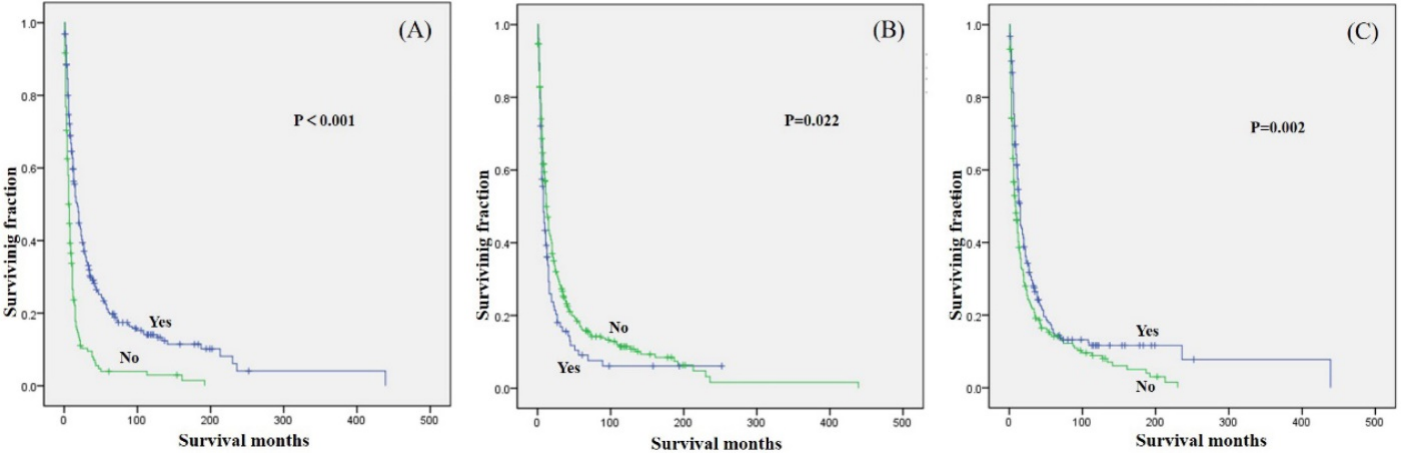
Kaplan-Meier method estimated OS in secondary osteosarcoma patients aged over 40 years stratified by (A) surgery, (B) radiation, and (C) chemotherapy.

Among secondary older osteosarcoma patients, only age, tumor site, tumor grade, tumor stage, tumor size, surgery (Fig.3A), radiation treatment (Fig.3B) and chemotherapy (Fig.3C) showed significant differences in OS.

### Multivariate analysis of independent predictors of OS or CSS in osteosarcoma patients aged over 40

The prognostic factors for older osteosarcoma patients are shown in Table 4. On multivariate analysis of all primary older osteosarcoma patients, age > 60, male, axial site, high grade, metastasis, tumor size > 10 cm, no surgery, and radiation treatment were negatively associated with OS. In terms of CSS, age, gender, decade of diagnosis, tumor site, tumor grade, tumor stage, tumor size, and surgery were independent prognostic factors. A multivariate Cox regression model showed that secondary older osteosarcoma patients of high grade, metastasis, tumor size > 10 cm, no surgery, and no chemotherapy were correlated with higher risk of mortality.

## Discussion

Osteosarcoma is the most frequent primary malignant bone tumor, and occurs predominantly in children and young adults 16, 17. Recently, the occurrence of older osteosarcoma patients has increased, and the prognosis of them is very poor 10. Because cases of osteosarcoma patients aged over 40 are rare, few studies have documented the prognostic factors of this special cohort 10. This is the largest study to report outcomes of older osteosarcoma patients, and the first to analyze the prognosis of primary and secondary osteosarcoma patients at the same time. The strengths of our study were its size (1162 primary older osteosarcoma patients and 444 secondary older osteosarcoma patients). We also analyzed the effects of controversial risk factor- radiotherapy on survival of older osteosarcoma patients.

Patients with osteosarcoma aged over 40 exhibit different clinical characteristics compared with children and young adults 18. Previous studies found that metastasis at presentation was more frequent in older patients than younger patients. Nearly a quarter of the patients in our study presented metastatic disease at diagnosis, which was higher than other studies 13, 19, 20 but similar with one previous report 10. Axial osteosarcoma occurred more frequently in the older than adolescents. Our study revealed that the incidence of axial osteosarcoma in primary older osteosarcoma patients was 19.4%, which was lower than that of previous studies 20, 21. Compared with the primary older osteosarcoma, secondary older osteosarcoma showed a predilection for axial sites (33.8%). Additionally, in our study, osteosarcoma, NOS was the most common histological type in both primary and secondary older osteosarcoma, which were similar to a previous study of all osteosarcoma 3.

Due to the frequent axial occurrence and metastasis in this age group, the outcome is usually poor. Iwata *et al.* 10 found that the 5-year OS and Event-free survival (EFS) in 86 osteosarcoma patients aged over 40 were 38% and 34%, respectively. Carsi *et al.* 21 reported that the 5-year OS and EFS rates were 41.64% and 32.54%, respectively. However, Ozkurt *et al.* 11 reported that the survival rate of the same age group was 76.2% at 2 years and 72.8% at 5 years in 36 cases. It is possible that their follow-up time is too short (median: 7 months) and treatment is more aggressive.

Ferrari *et al.* 12 reported that the 5-year OS of bone-sarcoma patents aged over 40 and synchronous metastases was 22%. In our study, the 3- and 5-year OS rates of the primary older osteosarcoma were 47.1% and 38.5%, respectively, while the 3- and 5-year OS rates of the secondary older osteosarcoma were 22.8% and 14.6%, suggesting a pretty poor outcome. Thus, it is necessary to explore prognostic factors to better guide the management of such patients.

Age younger than 60 years was considered as a prognostic factor for improved survival for primary older osteosarcoma but not for secondary older osteosarcoma. Male sex was identified as an independent risk factor for decreased OS and CSS. But for secondary older osteosarcoma, gender was not associated with OS. Many studies also identified male sex as a poor prognostic value for osteosarcoma 3, 22, 23. Frequent aggressive tumors or poorer response to treatment in males may account for it 3. An appendicular location of the osteosarcoma was associated with a better outcome compared with an axial location, which was similar with other reports 2, 3, 10. Tumor grade and stage were generally recognized as a very important predictor of osteosarcoma 3, 10.

Similarly, our study revealed that tumor grade and stage were independent prognostic factors of both OS and CSS. Many studies have reported that the tumor size above 10 cm is associated with poorer prognosis and decreased survival rate of osteosarcoma patients 7, 24, 25. In our cohort, tumor size > 10 cm was also an independent predictor of both OS and CSS.

Surgical excision and chemotherapy are considered the standard treatment strategy for osteosarcoma, but the efficacy of chemotherapy in older patients in particular is still controversial. Chemotherapy-related toxicity including peripheral neuropathy, hematological toxicity, and nephrotoxicity was as considerable and generally higher than those younger patients 12, 13. Therefore, treating the older osteosarcoma remains a challenge. Many authors hold that older osteosarcoma patients should receive aggressive chemotherapy and surgery if at all possible to achieve a survival rate similar to that observed in adolescents 11, 12, 26. However, Iwata *et al.* 10 reported that definitive surgery was a significant prognostic factor, whereas chemotherapy did not influence survival, which was similar to the results of primary older osteosarcoma patients in our study. Other studies also found chemotherapy did not prolong the survival of the older osteosarcoma patients 20, 21. However, among the secondary older osteosarcoma patients, surgical resection of primary tumors and systemic chemotherapy significantly prolonged the OS. Thus, surgery in combination with chemotherapy is recommended for the treatment of the secondary older osteosarcoma patients, while for the primary older osteosarcoma patients, only surgery is recommended.

Regarding radiotherapy, a satisfactory outcome is usually not achieved in osteosarcoma patients. Radiotherapy may offer local control as osteosarcoma is radioresistant 14. Its impact on survival of this age group remains controversial. Some studies reported that radiotherapy was associated with the prognosis in osteosarcoma 27-29. Schwarz *et al.* 28 found that the combination of surgery, chemotherapy, and radiotherapy was the optimum choice and the consistent use of full-dose chemotherapy is significant for the response to radiotherapy. However, Arshi *et al.* 30 found that radiation treatment was significantly associated with worse outcomes in patients with spinal osteosarcoma. Similarly, our study found that radiotherapy predicted worse OS and CSS of the primary older osteosarcoma, suggesting that radiation treatment is not an appropriate therapy for treating such patients. Among secondary older osteosarcoma patients, radiotherapy trended towards decreased survival but did not achieve statistical significance for OS on multivariate analysis. Additionally, many studies reported that radiation induced the occurrence of osteosarcoma with aggressive behaviors, suggesting that its use should be careful 31-33.

This study had several limitations. First, this study was a retrospective study from a large secondary database, which does not provide access to detailed clinical information. Prospective study should be performed to further confirm our conclusion. Second, the SEER database does not include other important information such as time to recurrence during follow-up, radiotherapy regimen and molecular pathological characteristics, which may affect the prognosis of patients. These variables may be an effective complement to this study, which will be an important section of our future research. Third, only 452(38.9%) of the primary osteosarcoma patients were older than 60-years, whereas 289(65.1%) of the secondary osteosarcoma patients were older than 60-years. Thus, there was age difference between the primary and secondary osteosarcoma patients. Age was an independent predictor of both OS and CSS in the primary older osteosarcoma patients. However, age was not an independent predictor of OS in the secondary older osteosarcoma patients. Thus, we think the OS and CSS rates between these two groups could be compared to a certain extent. But we also think more clinical researches should be done to get more convincing results. Despite these limitations, our large sample size along with demographic and tumor data allows for the investigation of important associations and predictors of older osteosarcoma. Additionally, the SEER database provides high statistical power due to the collection of data from multiple centers.

## Conclusion

We firstly and simultaneously analyzed the prognostic factors of 1162 primary older osteosarcoma patients and 444 secondary older osteosarcoma patients. Surgery in combination with chemotherapy is recommended for the treatment of the secondary older osteosarcoma patients, while for the primary older osteosarcoma patients, only surgery is recommended.

## Figures and Tables

**Table 1:** Demographic and clinical characteristics of primary osteosarcoma patients (n=1162) and secondary osteosarcoma patients (n=444) aged over 40 identified in the SEER database from 1973 to 2015

Variable	Primary	Secondary
*Age(years)*		
Mean	58	66
Median	56	67
≤60	710(61.1%)	155(34.9%)
>60	452(38.9%)	289(65.1%)
*Gender*		
Female	533(45.9%)	221(49.8%)
Male	629(54.1%)	223(50.2%)
*Decade of diagnosis*		
1970s	83(7.1%)	21(4.7%)
1980s	130(11.2%)	37(8.3%)
1990s	187(16.1%)	93(20.9%)
≥2000s^a^	762(65.6%)	293(66.0%)
*Race*		
White	914(78.7%)	362(81.5%)
Black	157(13.5%)	51(11.5%)
Other	91(7.8%)	31(7.0%)
*Location*		
Appendicular	637(54.8%)	149(33.6%)
Axial	225(19.4%)	150(33.8%)
Other locations	300(25.8%)	145(32.7%)
*Tumor type*		
Osteosarcoma, not otherwise specified (NOS)	836(71.9%)	291(65.5%)
Chondroblastic osteosarcoma	114(9.8%)	44(9.9%)
Fibroblastic osteosarcoma	89(7.7%)	24(5.4%)
Parosteal osteosarcoma	55(4.7%)	3(0.7%)
Telangiectatic osteosarcoma	24(2.1%)	3(0.7%)
Central osteosarcoma	17(1.5%)	6(1.4%)
Small cell osteosarcoma	11(0.9%)	1(0.2%)
Periosteal osteosarcoma	11(0.9%)	—
Intraosseous well differentiated osteosarcoma	3(0.3%)	1(0.2%)
High grade surface osteosarcoma	2(0.2%)	1(0.2%)
Osteosarcoma in Paget disease of bone	—	70(15.8%)
*Tumor grade^b^*		
Low	158(13.6%)	30(6.8%)
High	612(52.7%)	262(59.0%)
Unknown	392(33.7%)	152(34.2%)
*Tumor stage*		
Localized	354(30.5%)	115(25.9%)
Regional	456(39.2%)	178(40.1%)
Distant	264(22.7%)	106(23.9%)
Unknown	88(7.6%)	45(10.1%)
*Tumor size*		
<5cm	169(14.5%)	66(14.9%)
5-10cm	316(27.2%)	96(21.6%)
>10cm	239(20.6%)	74(16.7%)
Unknown	438(37.7%)	208(46.8%)
*Surgery*		
Yes	877(75.5%)	288(64.9%)
No	285(24.5%)	156(35.1%)
*Radiation treatment*		
Yes	272(23.4%)	104(23.4%)
No	890(76.6%)	340(76.6%)
*Chemotherapy*		
Yes	603(51.9%)	221(49.8%)
No	559(48.1%)	223(50.2%)
*Dead*		
Yes	781(67.2%)	376(84.7%)
No	381(32.8%)	68(15.3%)
*3-year OS rate*	47.1%	22.8%
*3-year CSS rate*	45.6%	—
*5-year OS rate*	38.5%	14.6%
*5-year CSS rate*	37.1%	—

a: 2000-2015 year; b: Low: ICD-O-3 Grade 1 (well differentiated) and Grade 2 (moderately differentiated); High: ICD-O-3 Grade 3 (poorly differentiated) and Grade 4 (undifferentiated anaplastic).

**Table 2:** Median survival data (month) of osteosarcoma patients aged over 40

Variable	Primary					Secondary	
	OS (estimate±SE )	95%CI	CSS (estimate±SE )	95%CI		OS (estimate±SE )	95%CI
*Overall*	30.0±2.6	24.8-35.2	28.0±2.4	23.4-32.6		12.0±0.9	10.3-13.7
*Age (years)*							
≤60	56.0±7.0	42.2-69.8	53.0±7.3	38.7-67.3		20.0±2.5	15.0-25.0
>60	14.0±1.3	11.5-16.5	12.0±1.0	10.0-14.0		9.0±0.9	7.3-10.7
*Gender*							
Female	36.0±5.3	25.6-46.4	35.0±4.9	25.5-44.5		11.0±1.4	8.3-13.7
Male	27.0±3.2	20.7-33.3	25.0±2.4	20.3-29.7		12.0±1.3	9.4-14.6
*Decade of diagnosis*							
1970s	14.0±2.9	8.3-19.7	10.0±1.9	6.2-13.8		11.0±2.8	5.4-16.6
1980s	18.0±2.9	12.4-23.6	14.0±2.7	8.7-19.3		9.0±1.5	6.1-11.9
1990s	36.0±8.8	18.7-53.3	23.0±6.0	11.3-34.7		17.0±4.0	9.1-24.9
≥2000s^a^	35.0±3.7	27.7-42.3	37.0±4.1	28.9-45.1		12.0±0.8	10.5-13.5
*Race*							
White	28.0±2.8	22.5-33.5	26.0±2.7	20.8-31.2		12.0±1.1	9.8-14.2
Black	37.0±8.0	21.3-52.7	29.0±7.0	15.3-42.7		12.0±2.2	7.7-16.3
Other	37.0±10.3	16.8-57.2	39.0±11.1	17.2-60.8		10.0±1.0	8.0-12.0
*Location*							
Appendicular	45.0±4.9	35.4-54.6	39.0±4.5	30.2-47.8		18.0±2.7	12.7-23.3
Axial	12.0±1.4	9.2-14.8	12.0±1.5	9.1-14.9		8.0±0.8	6.3-9.7
Other locations	40.0±8.3	23.7-56.3	38.0±7.9	22.6-53.4		13.0±1.0	11.0-15.0
*Tumor grade^b^*							
Low	191.0±22.6	146.8-235.3	NA	NA		41.0±40.2	0.000-119.7
High	27.0±2.8	21.5-32.5	26.0±2.8	20.5-31.5		13.0±1.1	10.8-15.2
*Tumor stage*							
Localized	124.0±26.7	71.6-176.4	241.0±74.5	94.9-387.1		19.0±3.3	12.5-25.5
Regional	42.0±6.0	30.2-53.8	37.0±5.0	27.1-46.9		16.0±1.7	12.7-19.3
Distant	7.0±0.7	5.6-8.4	7.0±0.8	5.4-8.6		6.0±0.8	4.5-7.5
*Tumor size*							
<5cm	160.0±37.6	86.4-233.6	NA	NA		22.0±2.7	16.6-27.4
5-10cm	52.0±9.2	34.0-70.0	52.0±10.3	31.8-72.2		19.0±2.5	14.2-23.8
>10cm	19.0±1.6	15.8-22.2	19.0±1.8	15.4-22.6		10.0±1.2	7.6-12.4
*Surgery*							
Yes	50.0±5.7	38.7-61.3	45.0±4.7	35.8-54.2		18.0±1.7	14.7-21.3
No	8.0±1.0	6.1-9.9	9.0±1.0	7.0-11.0		7.0±0.6	5.8-8.2
*Radiation treatment*							
Yes	14.0±1.2	11.6-16.4	14.0±1.4	11.2-16.8		8.0±1.2	5.6-10.4
No	46.0±4.7	36.8-55.2	41.0±4.7	31.7-50.3		12.0±1.0	10.0-14.0
*Chemotherapy*							
Yes	29.0±2.9	23.4-34.6	28.0±2.8	22.6-33.4		15.0±1.1	12.9-17.1
No	30.0±5.6	19.1-40.9	26.0±4.4	17.5-34.6		9.0±1.2	6.6-11.4

OS: overall survival, CSS: cancer-specific survival, N/A means that the median survival time was not available due to death event occurring in fewer than 50% of cases in the cohort. SE: standard error.

**Table 3:** Univariate analyses of variables in osteosarcoma patients aged over 40 using Kaplan-Meier method

Variable	Primary			Secondary
	OS (Log-rank p value)	CSS (Log-rank p value)		OS (Log-rank p value)
*Age* (≤60 vs>60)	<0.001	<0.001		<0.001
*Gender*	0.013	0.007		0.750
*Decade of diagnosis*	<0.001	<0.001		0.200
≥2000sa vs1970s	<0.001	<0.001		—
≥2000sa vs 1980s	0.002	<0.001		—
≥2000sa vs 1990s	0.791	0.058		—
1990s vs 1970s	0.006	<0.001		—
1990s vs 1980s	0.050	0.004		—
1980s vs 1970s	0.224	0.004		—
*Race*	0.757	0.430		0.396
*Location*	<0.001	<0.001		<0.001
Axial vs Appendicular	<0.001	<0.001		<0.001
Axial vs Other locations	<0.001	<0.001		0.001
Appendicular vs Other locations	0.938	0.861		0.408
*Tumor grade^b^* (Low vs High)	<0.001	<0.001		0.001
*Tumor stage*	<0.001	<0.001		<0.001
Distant vs Localized	<0.001	<0.001		<0.001
Distant vs Regional	<0.001	<0.001		<0.001
Regional vs Localized	<0.001	<0.001		0.252
*Tumor size*	<0.001	<0.001		<0.001
>10cm vs <5cm	<0.001	<0.001		<0.001
>10cm vs 5-10cm	<0.001	<0.001		0.001
5-10cm vs <5cm	0.001	0.003		0.235
*Surgery*	<0.001	<0.001		<0.001
*Radiation treatment*	<0.001	<0.001		0.022
*Chemotherapy*	0.581	0.742		0.002

**Table 4:** Multivariate analyses for OS and CSS for osteosarcoma patients aged over 40

Variable	Primary					Secondary	
	OS		CSS			OS	
	Hazard Ratio(95% CI)	P value	Hazard Ratio(95% CI)	P value		Hazard Ratio(95% CI)	P value
*Age (years)*							
≤60	1		1			1	
>60	1.887(1.624-2.194)	<0.001	1.927(1.622-2.288)	<0.001		1.251(0.981-1.596)	0.071
*Gender*							
Female	1		1			—	
Male	1.185(1.023-1.373)	0.024	1.181(1.002-1.392)	0.047		—	—
*Decade of diagnosis*							
1970s	1		1			—	
1980s	1.099(0.819-1.476)	0.528	0.855(0.607-1.206)	0.373		—	—
1990s	0.870(0.649-1.168)	0.354	0.642(0.456-0.903)	0.011		—	—
≥2000s^a^	0.804(0.612-1.057)	0.119	0.526(0.387-0.715)	<0.001		—	—
*Location*							
Appendicular	1		1			1	
Axial	1.708(1.418-2.057)	<0.001	1.748(1.427-2.143)	<0.001		1.280(0.979-1.673)	0.071
Other locations	1.066(0.879-1.292)	0.517	1.100(0.887-1.365)	0.385		1.209(0.920-1.589)	0.172
*Tumor grade^b^*							
Low	1		1			1	
High	2.197(1.653-2.919)	<0.001	2.682(1.906-3.775)	<0.001		1.871(1.141-3.070)	0.013
*Tumor stage*							
Localized	1		1			1	
Regional	1.174(0.967-1.425)	0.106	1.252(0.997-1.573)	0.053		1.176(0.893-1.548)	0.250
Distant	2.904(2.322-3.631)	<0.001	3.195(2.479-4.119)	<0.001		2.678(1.936-3.704)	<0.001
*Tumor size*							
<5cm	1		1			1	
5-10cm	1.268(0.965-1.666)	0.089	1.280(0.939-1.744)	0.118		1.179(0.804-1.729)	0.399
>10cm	1.699(1.264-2.283)	<0.001	1.727(1.242-2.402)	0.001		1.778(1.161-2.724)	0.008
*Surgery*							
Yes	1		1			1	
No	1.604(1.333-1.931)	<0.001	1.460(1.194-1.785)	<0.001		1.405(1.080-1.829)	0.011
*Radiation treatment*							
Yes	1		1			1	
No	0.716(0.601-0.852)	<0.001	1.247(1.025-1.518)	0.027		0.898(0.697-1.157)	0.405
*Chemotherapy*							
Yes	—		—			1	
No	—	—	—	—		1.337(1.066-1.678)	0.012
